# Predictors of In-Hospital Mortality and Incomplete Kidney Recovery in Patients with Cirrhosis-Associated Non-Hepatorenal Syndrome Acute Kidney Injury: A Single-Center Retrospective Cohort Study

**DOI:** 10.3390/jcm15176673

**Published:** 2026-08-28

**Authors:** Daniela Rădulescu, Ileana Adela Văcăroiu, Andreea Manuela Franculescu-Bertea, Alex Nicolae Șendrescu, Alexandra Elisabeta Matea-Moldovan, Flavia Liliana Turcu, Daiana Cristina Brehui-Bertea

**Affiliations:** 1 Clinical Department 3—Nephrology Discipline, Faculty of Medicine, Carol Davila University of Medicine and Pharmacy, 020021 Bucharest, Romania; ileana.vacaroiu@umfcd.ro (I.A.V.); alex-nicolae.sendrescu25@rez.umfcd.ro (A.N.Ș.); alexandra-elisabeta.matea-moldovan@rez.umfcd.ro (A.E.M.-M.); flavia.turcu@umfcd.ro (F.L.T.); cristina-daiana.brehui-bertea@rez.umfcd.ro (D.C.B.-B.); 2 Department of Nephrology, Sf. Ioan Clinical Emergency Hospital, 042122 Bucharest, Romania; 3 Clinical Department 3—Internal Medicine Discipline, Faculty of Medicine, Carol Davila University of Medicine and Pharmacy, 020021 Bucharest, Romania; andreea.franculescu-bertea@umfcd.ro; 4 Department of Internal Medicine, Fundeni Clinical Institute, 022328 Bucharest, Romania

**Keywords:** liver cirrhosis, non-hepatorenal syndrome-acute kidney injury, renal recovery, mortality, C-reactive protein

## Abstract

**Background**: Acute kidney injury (AKI) is associated with poor outcomes in patients with cirrhosis. However, most studies evaluate AKI as a single clinical entity, whereas predictors of mortality and renal recovery, specifically in non-hepatorenal syndrome AKI (non-HRS-AKI), remain poorly defined. **Methods**: We performed a retrospective cohort study including consecutive patients with liver cirrhosis admitted with non-HRS-AKI to a multidisciplinary emergency hospital between 1 January 2024 and 31 December 2025. Only variables available at hospital admission were included in the multivariable analyses. Independent predictors of in-hospital mortality and factors associated with incomplete renal recovery in survivors at hospital discharge were identified using multivariable logistic regression. **Results**: A total of 139 patients were included. Overall, 57 patients (41.0%) died during hospitalization. Among the 82 survivors, complete renal recovery occurred in 35 cases (42.7%), whereas 47 patients (57.3%) had incomplete renal recovery at discharge. Independent predictors of in-hospital mortality were a higher MELD-Na score (adjusted OR 1.16 per 1-point increase, 95% CI 1.07–1.26; *p* < 0.001), an advanced AKI stage (KDIGO stage 3 vs. stage 1, adjusted OR 6.71, 95% CI 1.73–26.04; *p* = 0.006), the absence of pre-existing chronic kidney disease (adjusted OR 0.233, 95% CI 0.085–0.644; *p* = 0.005), and higher admission C-reactive protein (adjusted OR 1.12 per 10 mg/L increase, 95% CI 1.01–1.23; *p* = 0.029). Factors independently associated with incomplete renal recovery among hospital survivors at hospital discharge were pre-existing heart failure (adjusted OR 3.07, 95% CI 1.10–8.62; *p* = 0.033) and higher admission C-reactive protein (adjusted OR 1.17 per 10 mg/L increase, 95% CI 1.00–1.34; *p* = 0.047). **Conclusions**: In patients with cirrhosis-associated non-HRS-AKI, in-hospital mortality was primarily associated with the severity of liver disease and AKI, whereas incomplete renal recovery at hospital discharge was independently associated with pre-existing heart failure. Admission C-reactive protein was independently associated with both outcomes, supporting the role of systemic inflammation in determining short-term prognosis and suggesting that C-reactive protein may serve as a simple, readily available biomarker for early risk stratification.

## 1. Introduction

Acute kidney injury (AKI) is a frequent complication in patients with liver cirrhosis [1,2] and is strongly associated with poor outcomes, including increased short-term mortality, higher rates of multi-organ failure, and prolonged intensive care unit admission [3,4]. Incomplete kidney recovery after AKI and progression toward chronic kidney disease (CKD) have increasingly been recognized in patients with cirrhosis and are associated with adverse long-term complications and reduced eligibility for liver transplantation [5,6].

The diagnostic criteria for AKI in cirrhosis were updated in 2024 by Acute Disease Quality Initiative (ADQI) and International Club of Ascites (ICA) joint multidisciplinary consensus meeting [7], and they are largely consistent with the KDIGO (Kidney Disease: Improving Global Outcomes) criteria [8], i.e., an increase in serum creatinine (SCr) ≥ 0.3 mg/dL (26.5 μmol/L) within 48 h or ≥50% from the baseline value known or presumed to have occurred within the prior 7 days and/or urine output (UO) ≤ 0.5 mL/kg for ≥6 h.

AKI in cirrhosis is a heterogeneous entity encompassing both functional and structural renal injury. While hepatorenal syndrome–AKI (HRS-AKI) represents a unique entity specific only to cirrhosis [9], a substantial proportion of cases are classified as non-HRS-AKI, including prerenal azotemia, acute tubular necrosis and other intrinsic renal diseases, which may differ significantly in pathophysiology and prognosis [7].

Despite the well-established prognostic impact of AKI in cirrhosis, the current evidence is predominantly focused on HRS-AKI, while non-HRS-AKI remains under-investigated and poorly characterized in terms of clinical predictors of mortality. The existing studies often group heterogeneous AKI phenotypes together or exclude non-HRS subtypes, limiting the ability to accurately stratify risk and guide clinical management. Consequently, predictors of adverse outcomes specifically in patients with non-HRS-AKI remain insufficiently characterized.

Therefore, the primary aim of the present study was to identify predictors of in-hospital mortality in patients with cirrhosis admitted with non-HRS-AKI. The secondary objective was to identify factors associated with incomplete kidney recovery at hospital discharge among survivors. Improved identification of patients at increased risk of death or persistent renal dysfunction may facilitate early risk stratification and optimize clinical management.

## 2. Materials and Methods

This retrospective cohort study included adult patients admitted to a multidisciplinary emergency hospital between 1 January 2024 and 31 December 2025 with concomitant liver cirrhosis (LC) and non-hepatorenal syndrome acute kidney injury (non-HRS-AKI) during the same hospitalization episode. As this was a retrospective exploratory cohort study including all eligible patients identified during the predefined study period, no a priori sample-size calculation was performed.

**Figure 1:**
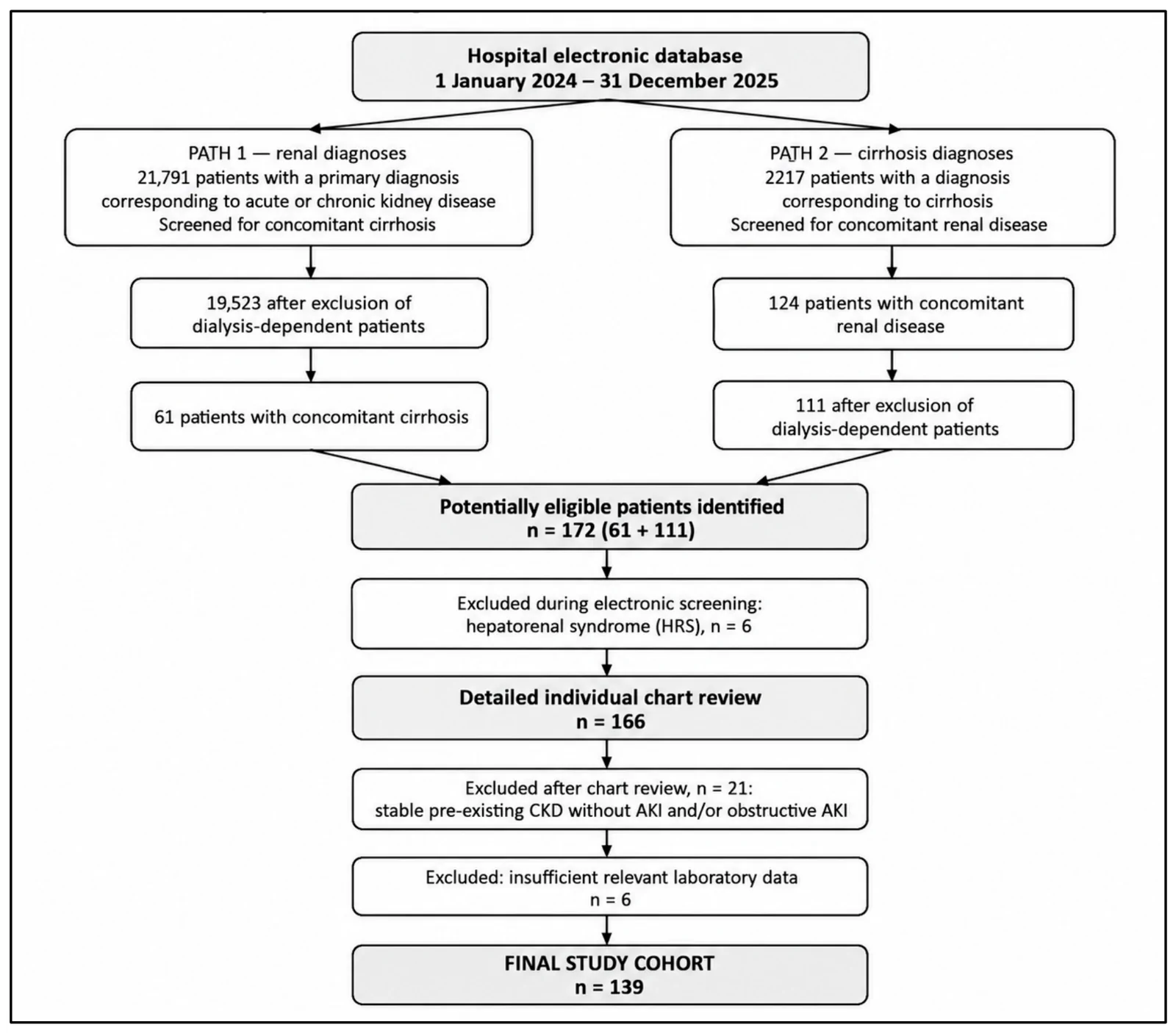
Patient identification and cohort selection. STROBE-style flow diagram illustrating the electronic database screening, the two complementary patient-identification pathways, subsequent exclusions, individual chart review, and selection of the final study cohort. CKD, chronic kidney disease; AKI, acute kidney injury; HRS, hepatorenal syndrome.

Patients were identified from the hospital electronic database using ICD-10 discharge diagnoses corresponding to acute or chronic kidney disease and liver cirrhosis during the study period (1 January 2024 to 31 December 2025). To maximize case ascertainment, two complementary screening pathways were used: patients with a primary renal diagnosis were screened for concomitant cirrhosis, and patients with a primary cirrhosis diagnosis were screened for concomitant renal disease. Patients with dialysis dependence were excluded during electronic screening. This process identified 172 potentially eligible patients. Six patients with a documented diagnosis of hepatorenal syndrome (HRS) were subsequently excluded during the electronic screening of secondary diagnoses, leaving 166 patients for detailed individual chart review. A manual review subsequently excluded 21 patients who did not meet the study eligibility criteria, including patients with stable pre-existing CKD without an acute kidney injury episode and patients with obstructive AKI, as well as six patients with insufficient relevant laboratory data. The final cohort therefore comprised 139 patients (Figure 1). Eligibility assessment and AKI classification were based on an individual chart review, as described below. As all laboratory variables included in the analyses were routinely obtained at hospital admission, missing data were minimal, and only a few patients were excluded because of incomplete baseline information.

AKI was defined and staged according to KDIGO criteria, in accordance with the ADQI and ICA consensus recommendations [7,8]. Baseline serum creatinine was defined as the most recent pre-hospitalization value within the 12 months preceding admission.

Recurrent AKI episodes were included only if occurring at least 3 months apart. In cases of multiple AKI episodes during the same hospitalization, only the first episode was analyzed.

Pre-existing chronic kidney disease (CKD) was defined as a documented estimated glomerular filtration rate (eGFR) < 60 mL/min/1.73 m^2^ persisting for at least 3 months within the 12 months preceding hospitalization. eGFR was calculated using the CKD-EPI equation based on serum creatinine values obtained from outpatient or previous hospital records.

AKI etiology was determined through detailed retrospective clinical adjudication based on the integrated clinical, laboratory, and urinary information available at hospital admission. All relevant potential precipitating factors identified during chart review were recorded in the study database, and individual patients could have more than one potential contributor to AKI. For the primary etiological classification, however, each patient without HRS was assigned a single predominant AKI phenotype, based on the cause considered most likely to have primarily contributed to the AKI episode. The assessment integrated hemodynamic and volume status, infection/sepsis, documented nephrotoxic exposures, laboratory findings, urinary indices when available, and other relevant clinical information. Cases were initially screened by a resident physician and independently reviewed by two senior physicians, with final classification established by consensus. This etiological classification was independent of the discharge diagnosis and was not modified according to in-hospital renal recovery. Patients with evidence of toxic tubular injury related to documented nephrotoxic exposures, including intravenous contrast or nephrotoxic antimicrobial therapy, were classified as intrinsic AKI. Other intrinsic etiologies included ischemic acute tubular necrosis, rhabdomyolysis-associated kidney injury, acute tubulointerstitial nephritis, and other documented intrinsic renal diseases. In patients with sepsis, the predominant clinical phenotype determined classification as sepsis-associated prerenal AKI or sepsis-associated intrinsic AKI (sepsis-associated ATN).

Renal recovery in survivors was assessed at hospital discharge and categorized as follows:

- Complete recovery: serum creatinine returned to within 0.3 mg/dL of baseline
- Incomplete recovery: failure to meet this criterion or dependence on chronic dialysis at hospital discharge.

The prognostic significance of the initial AKI phenotype was evaluated by analyzing renal recovery outcomes according to admission-based classification.

Severity of liver dysfunction was assessed using the Child–Pugh classification, which was retrieved from chart documentation when available, or otherwise calculated by a gastroenterologist from its five standard components; the MELD-Na score was calculated using admission laboratory parameters. The etiology of cirrhosis was also recorded from clinical charts.

Demographic data, comorbidities, characteristics of cirrhosis and AKI, and laboratory parameters at admission (including serum hemoglobin, platelet count, urea, creatinine, sodium, albumin, INR, and C-reactive protein), as well as in-hospital outcomes such as intensive care unit (ICU) admission, requirement for acute hemodialysis (HD), recovery of kidney function in survivors and mortality were collected and analyzed. Comorbidities were identified from documented diagnoses in the patients’ medical records and/or discharge diagnoses and ICD-10 coding.

All data were reviewed and validated prior to statistical analysis to ensure consistency and accuracy of clinical classification for both AKI and LC.

The study was approved by the Institutional Ethics Committee (approval no. R20817/9 December 2025). The requirement for informed consent was waived because of the retrospective design of the study.

Statistical analysis was performed using Jamovi software (version 2.7.34). Continuous variables were assessed for normality of distribution using graphical methods and the Shapiro–Wilk test. As most continuous variables showed non-normal distributions, they were expressed as median and interquartile range (IQR) and compared between groups using the Mann–Whitney U test.

Categorical variables were summarized as absolute numbers and percentages. Comparisons between survivors and non-survivors were performed using Pearson’s chi-square test. Fisher’s exact test was applied when the expected frequency in one or more contingency table cells was less than five.

Variables considered clinically relevant and/or associated with the outcome in univariable analyses were considered for multivariable logistic regression. Variable selection was not based solely on statistical significance; clinical relevance, biological plausibility, and potential overlap between related variables were also considered. Given the limited number of outcome events, model complexity was restricted to reduce the risk of overfitting. Alternative multivariable models were explored when clinically related or potentially overlapping predictors required separate evaluation.

Multivariable logistic regression models are reported using adjusted odds ratios (ORs) with 95% confidence intervals (CIs) and *p*-values. Multicollinearity was assessed using variance inflation factors (VIFs). Model calibration was assessed using the Hosmer–Lemeshow goodness-of-fit test, discrimination using the area was assessed under the receiver operating characteristic curve (AUC/C-statistic), and internal validation was assessed using bootstrap resampling with 1000 samples. Bootstrap validation was used to estimate mean optimism and the optimism-corrected AUC.

All statistical tests were two-sided, and a *p*-value < 0.05 was considered statistically significant.

## 3. Results

A total of 139 patients were included in the study. Baseline characteristics are presented in Table 1 and Table 2. Most patients were male (79.1%), and alcohol-associated liver disease represented the predominant etiology of cirrhosis. Almost 80% of the patients were classified as having Child–Pugh class B and C of cirrhosis, and the median MELD-sodium score at admission was 26. Regarding AKI etiology, volume depletion dominated prerenal AKI, while in the group of intrinsic AKI, most cases were secondary to sepsis. Previous CKD was present in about half of the patients. Seven patients had no documented baseline serum creatinine within the preceding 12 months; all had an admission serum creatinine >4 mg/dL and were therefore classified as KDIGO stage 3.

During hospitalization, 57 patients (41%) died. Baseline characteristics according to in-hospital survival are summarized in Table 3. Compared with survivors, patients who died were younger and had significantly higher MELD-Na scores, more advanced liver disease (Child–Pugh class C), lower serum albumin levels, more severe AKI (KDIGO stage 3), a higher prevalence of intrinsic AKI, and higher admission C-reactive protein levels. Conversely, AKI superimposed on pre-existing CKD was more frequent among survivors. During hospitalization, ICU admission occurred almost exclusively among non-survivors.

In univariable logistic regression analysis (Table 4), higher MELD-Na score, Child–Pugh class C, KDIGO stage 2 and 3 AKI, intrinsic AKI, sepsis-associated AKI, higher C-reactive protein levels, lower serum albumin levels, and the need for acute hemodialysis were significantly associated with increased odds of in-hospital mortality. AKI superimposed on pre-existing CKD was associated with lower odds of death. Variables considered clinically relevant together with those significant in univariable analysis were entered into the multivariable logistic regression model.

Twelve candidate variables were considered for multivariable modeling based on clinical relevance and/or findings from univariable analysis. Variable selection was guided by clinical considerations, potential redundancy between predictors, and model parsimony rather than by statistical significance alone. The primary parsimonious model (Model A) included MELD-Na, KDIGO stage, pre-existing CKD, and admission C-reactive protein (Table 5). Additional sensitivity models (Table 5) were constructed to assess the independent contribution of sepsis and intrinsic AKI (Models B–D).

In the primary model (Model A), higher MELD-Na score, higher admission C-reactive protein, and more advanced AKI stage (KDIGO stages 2 and 3) were independently associated with increased in-hospital mortality, whereas AKI superimposed on pre-existing CKD was independently associated with lower odds of death. No evidence of multicollinearity was observed among variables included in the primary model (all VIF values < 1.3).

Model A showed good discrimination, with an apparent AUC of 0.906. Its AIC was 117 and its McFadden R^2^ was 0.440. Calibration was acceptable according to the Hosmer–Lemeshow goodness-of-fit test (χ^2^ = 12.50, df = 8, *p* = 0.130). Internal validation using 1000 bootstrap samples yielded a mean optimism of 0.015, resulting in an optimism-corrected AUC of 0.891.

The sensitivity models showed AUCs of 0.897, 0.931, and 0.935 for Models B, C, and D, respectively. Their corresponding AIC values were 120, 102, and 104, and their McFadden R^2^ values were 0.424, 0.520, and 0.531, respectively. Models C and D showed higher apparent discrimination and lower AIC values than the primary model, but included intrinsic AKI, and were therefore considered sensitivity analyses rather than replacements for the primary parsimonious model.

A sensitivity analysis for primary model A excluding the seven patients without a documented baseline serum creatinine yielded similar results (*n* = 132). KDIGO stage 2 versus stage 1 remained significantly associated with in-hospital mortality (OR 4.52, 95% CI 1.23–16.63; *p* = 0.023), as did KDIGO stage 3 versus stage 1 (OR 8.55, 95% CI 1.94–37.68; *p* = 0.005).

Given the inverse association between pre-existing CKD and in-hospital mortality, an additional descriptive comparison was performed between patients with and without pre-existing CKD. Mortality was 25.7% (18/70) among patients with pre-existing CKD compared with 56.5% (39/69) among those without CKD. Patients with pre-existing CKD had a significantly lower median MELD-Na score at admission than patients without CKD (22 vs. 29, *p* = 0.012). Median C-reactive protein was also lower in patients with pre-existing CKD (39 vs. 48.9 mg/L), although this difference was not statistically significant (*p* = 0.211). The distribution of AKI severity according to KDIGO stage did not differ significantly between the two groups (*p* = 0.19).

Among the 82 survivors, 35 (42.7%) achieved complete renal recovery, whereas 47 (57.3%) had incomplete renal recovery at hospital discharge. Patients with incomplete renal recovery had a significantly longer hospital stay and were more likely to have chronic heart failure, Child–Pugh class C cirrhosis, more severe AKI (higher KDIGO stage), and more advanced pre-existing CKD (eGFR < 45 mL/min/1.73 m^2^). They also presented with higher admission C-reactive protein levels and lower serum albumin concentrations. Notably, none of the patients with intrinsic AKI achieved complete renal recovery. No significant differences were observed regarding age, sex, diabetes mellitus, hypertension, coronary artery disease, advanced pulmonary disease, history of stroke, malignancy, MELD-Na score, sepsis, hemoglobin, urea, or platelet count (Table 6).

Univariable logistic regression identified pre-existing heart failure, previous advanced CKD (eGFR < 45 mL/min/1.73 m^2^), lower serum albumin, higher admission C-reactive protein, and KDIGO stage 2 as candidate factors associated with incomplete kidney recovery at hospital discharge (Table 7).

After multivariable adjustment, pre-existing heart failure and admission C-reactive protein remained independently associated with incomplete kidney recovery. Patients with heart failure had approximately threefold higher odds of incomplete renal recovery at hospital discharge (adjusted OR 3.07, 95% CI 1.10–8.62, *p* = 0.033). In addition, every 10 mg/L increase in C-reactive protein at admission was associated with an approximately 17% higher odds of incomplete kidney recovery (adjusted OR 1.17, 95% CI 1.00–1.34, *p* = 0.047) (Table 8).

Nevertheless, several clinically plausible multivariable model specifications were explored. KDIGO stage 2 versus stage 1 was significantly associated with incomplete renal recovery in more than one model; however, KDIGO stage 3 was not, and estimates became unstable in more extensively adjusted models. Pre-existing heart failure showed the most consistent independent association across the clinically plausible models, while admission C-reactive protein remained independently associated in the parsimonious model.

## 4. Discussion

This retrospective cohort study investigated predictors of in-hospital mortality and factors associated with incomplete kidney recovery in patients with cirrhosis admitted with non-HRS acute kidney injury, exclusively using variables available at hospital admission. Although numerous studies have examined AKI in cirrhosis, most have analyzed it as a single clinical entity without distinguishing between different AKI phenotypes. In addition, comparisons across studies are limited by substantial variability in the diagnostic criteria and staging systems used to define AKI. By focusing exclusively on patients with non-HRS-AKI diagnosed according to contemporary consensus criteria, we sought to reduce this heterogeneity and provide a more homogeneous assessment of predictors of clinically relevant outcomes.

Our cohort was characterized by a high prevalence of alcohol-related advanced cirrhosis, consistent with the epidemiological profile of patients previously reported at our institution [10]. Prerenal and intrinsic AKI occurred with comparable frequencies, while approximately half of the patients had pre-existing CKD. These findings likely reflect the referral pattern of our institution rather than the epidemiology of cirrhosis-associated AKI in the general population. Our hospital is a multidisciplinary emergency referral center with dedicated gastroenterology and nephrology departments managing a large volume of acute and complex cases. The gastroenterology department provides advanced care for patients with decompensated cirrhosis, including emergency therapeutic endoscopy and other specialized interventions, reflecting the complexity of the hepatology cases referred to our institution [11]. In parallel, the nephrology department serves as the regional referral center for severe AKI and advanced CKD and is the only provider of emergency dialysis on a 24/7 basis. Consequently, our study population was enriched with patients presenting with advanced liver disease and severe kidney dysfunction, thereby introducing an inherent selection bias.

Overall, 57 of the 139 patients (41%) died during hospitalization. This mortality rate is consistent with previously published data. Recent meta-analyses reported pooled short-term mortality rates of approximately 34% among patients with cirrhosis and AKI, with substantially higher mortality observed in critically ill patients and in those with acute-on-chronic liver failure [4,12]. Nevertheless, mortality estimates vary according to the clinical setting, AKI definition, and severity of underlying liver disease, making direct comparisons across studies challenging [4,12]. Our results indicate that non-HRS-AKI carries a substantial risk of in-hospital death despite the exclusion of hepatorenal syndrome.

In the multivariable analysis, in-hospital mortality was independently associated with a higher MELD-Na score, more severe AKI (higher KDIGO stage) and elevated admission C-reactive protein levels. Conversely, AKI superimposed on pre-existing CKD was associated with lower odds of death.

The finding that higher admission MELD-Na score was an independent predictor of mortality in our cohort confirms the central role of global liver disease severity in determining short-term outcomes among patients with cirrhosis and non-HRS-AKI. MELD-Na incorporates serum bilirubin, creatinine, international normalized ratio (INR), and serum sodium, thereby simultaneously reflecting hepatic, renal and circulatory dysfunction. Because it captures multiple pathophysiological mechanisms involved in decompensated cirrhosis, MELD-Na has consistently demonstrated superior prognostic performance compared with isolated laboratory parameters [13,14]. In order to avoid multicollinearity, we deliberately did not include any MELD-Na components in the multivariable model. Our result is in agreement with previous studies demonstrating that increasing liver disease severity is strongly associated with mortality in patients with AKI [15,16,17], although none of these studies evaluated non-HRS-AKI separately. The revised International Club of Ascites (ICA) recommendations emphasized that AKI should always be interpreted in the context of overall hepatic dysfunction rather than as an isolated renal event [18]. More recently, the HRS-HARMONY Consortium demonstrated that, among patients requiring renal replacement therapy, the severity of liver disease rather than the specific AKI phenotype was the principal determinant of survival [19]. Interestingly, although Child–Pugh class C was strongly associated with mortality in univariable analysis, it did not remain an independent predictor after adjustment. This suggests that traditional measures of liver disease severity may be insufficient to fully capture the risk of death in this population. Taken together, these observations support our finding that MELD-Na remains a robust predictor of mortality even after excluding patients with HRS-AKI.

AKI severity according to KDIGO stage was independently associated with in-hospital mortality in our cohort. Compared with stage 1, patients with stage 2 and stage 3 AKI had a fourfold and sixfold higher odds of death, respectively, demonstrating a clear severity-dependent gradient. This finding is consistent with the results from numerous studies showing that increasing AKI stage is associated with progressively higher short-term mortality in patients with cirrhosis [12,20]. More advanced AKI stages likely reflect both greater renal injury and more severe systemic illness, including circulatory dysfunction, inflammation, sepsis, and multiorgan failure, all of which contribute to adverse outcomes in decompensated cirrhosis. The prognostic value of KDIGO staging persisted despite the exclusion of HRS-AKI, suggesting that the severity of renal dysfunction remains a major determinant of outcome across non-HRS phenotypes of cirrhosis-associated AKI.

Unexpectedly, patients with pre-existing CKD had approximately one quarter of the odds of in-hospital death compared with patients without CKD. Although this finding appears counterintuitive, it is supported by recent data from the HRS-HARMONY Consortium, which also demonstrated lower adjusted mortality among patients with AKI on CKD than among those with AKI alone [21]. One proposed explanation was that patients with AKI without pre-existing CKD had more severe underlying liver dysfunction, whereas those with AKI with pre-existing CKD more frequently presented with relatively preserved hepatic function despite worse baseline renal function [21]. In our cohort, patients with pre-existing CKD had a significantly lower median MELD-Na score at admission than those without pre-existing CKD (22 vs. 29, *p* = 0.012), supporting the possibility that differences in underlying liver disease severity contributed to the observed association. Yet, the inverse association persisted after adjustment for MELD-Na, suggesting that differences in hepatic disease severity alone do not fully explain the finding. The referral pattern of our institution may also have contributed, as many patients with pre-existing CKD were already under nephrology follow-up before admission, potentially allowing earlier recognition of renal deterioration and more timely nephrology-directed management. However, previous nephrology follow-up and timing of nephrology assessment were not systematically recorded and this hypothesis could not be directly tested. Survivor bias and collider bias related to selection into a tertiary-care cohort of patients with both cirrhosis and AKI also cannot be excluded, and residual confounding remains possible. Therefore, this result in our study should be interpreted as an observational inverse association rather than as evidence of a protective causal effect of pre-existing CKD.

We also revealed C-reactive protein at hospital admission as an independent predictor of in-hospital mortality in patients with non-HRS-AKI. Each 10 mg/L increase in C-reactive protein was associated with an approximately 12% increase in the odds of in-hospital mortality. This finding is biologically plausible, as both cirrhosis and acute kidney injury are characterized by systemic inflammation and immune dysregulation [22,23], processes that substantially contribute to organ failure and mortality [24,25]. Previous studies have consistently shown that elevated C-reactive protein predicts short-term mortality in patients with cirrhosis and AKI independently of conventional liver severity scores [20]. A recent analysis of the eICU Collaborative Research Database identified patients with sepsis-associated AKI, acute tubular necrosis, and hepatorenal syndrome as a high-inflammatory phenotype associated with increased mortality [26]. In our cohort, both sepsis-associated AKI and intrinsic AKI were more frequent among non-survivors (Table 3). However, after adjustment, sepsis was not independently associated with mortality, while the inclusion of intrinsic AKI attenuated the association between KDIGO stage and mortality. These findings support admission C-reactive protein as an independent marker of inflammatory burden in this population.

An interesting finding was the younger age of non-survivors in the univariable analysis. However, age was no longer independently associated with mortality after adjustment for liver disease severity, AKI stage, inflammatory status and pre-existing CKD. This suggests that the apparent effect of age was largely driven by the greater severity of illness among younger patients at presentation rather than by age itself. Similar observations have been reported in recent studies showing that the impact of AKI on mortality may have a stronger relative effect in younger patients [27].

In our cohort, among 82 patients who survived, 35 (42.7%) achieved complete renal recovery, whereas 47 (57.3%) had incomplete recovery at hospital discharge. Multivariable logistic regression identified heart failure and C-reactive protein at admission as factors independently associated with incomplete recovery, while previous severe CKD (eGFR < 45 mL/min/1.73 m^2^), AKI stage and serum albumin were associated with incomplete recovery in univariable analyses.

Although KDIGO stage 2 was associated with incomplete recovery in several model specifications, the absence of a corresponding association for stage 3 and the marked imprecision of estimates in more extensively adjusted models suggest that this finding should be interpreted cautiously in view of the small number of patients in the individual KDIGO categories. We therefore favored a parsimonious model in which the association of pre-existing heart failure was consistent and admission C-reactive protein remained independently associated with incomplete recovery.

In our study, none of the patients with intrinsic AKI achieved complete renal recovery. Nevertheless, this finding should be interpreted as a descriptive observation only, as the limited sample size and complete separation of outcomes did not permit reliable adjusted or causal inference regarding the association between intrinsic AKI and renal recovery.

Compared with mortality, renal recovery after AKI in cirrhosis has received considerably less attention, although there is evidence that cirrhotic patients are at high risk for maladaptive repair of AKI [5]. Most available studies have focused on medium- and long-term renal outcomes, such as acute kidney disease or progression to chronic kidney disease, rather than recovery at hospital discharge [3,28,29]. The few available studies addressing renal recovery evaluated highly selected clinical settings, such as contrast-induced AKI or stage 1B AKI [30,31]. An additional limitation of the current literature is that renal recovery has rarely been analyzed separately according to AKI phenotype. Most studies combine HRS-AKI and non-HRS-AKI into a single cohort despite their distinct pathophysiological mechanisms [20]. We identified only one study that classifies outcome in HRS vs. non-HRS-AKI, but the authors have evaluated the kidney function 3 months after hospitalization for AKI in cirrhotic patients [28].

In the present study, pre-existing chronic heart failure (CHF) emerged as the most important factor independently associated with incomplete kidney recovery after multivariable adjustment. Given recent advances in our understanding of the kidney–heart cross-talk in AKI and cardiorenal syndromes [32], this result is plausible and consistent with previous studies [33]. Beyond its effect on systemic hemodynamics, heart failure contributes to persistent renal hypoperfusion, increased central venous pressure and impaired renal venous drainage, mechanisms that may limit renal recovery even after resolution of the acute precipitating event. Nevertheless, in our cohort, the prevalence of heart failure was more than twofold higher among patients without complete renal recovery, but prevalence of comorbid predisposing conditions (i.e., CAD, HT, and diabetes) was not significantly different in comparison with the complete recovery group. Also, the prevalence of CKD, a major risk factor for heart failure [34], was not significantly different. Therefore, we cannot exclude the possibility that cirrhotic cardiomyopathy contributed to the higher prevalence of heart failure among patients without complete renal recovery. Cirrhotic cardiomyopathy is defined as a cardiac dysfunction in the absence of other cause, and it may coexist with CHF of another etiology [35]. It affects about 50% of patients, being more prevalent in severe forms of cirrhosis [36]. In the pathogenesis of cirrhotic cardiomyopathy, inflammation occupies a central role besides protein/lipid synthetic/metabolic defects [35], and there are data revealing that increased levels of C-reactive protein, a marker of inflammation, are associated with more severe forms of cirrhotic cardiomyopathy [37].

The second factor independently associated with incomplete recovery of kidney function in our study was C-reactive protein. C-reactive protein is synthesized by the liver and it would be expected to decrease in advanced cirrhosis. Yet, increasing C-reactive protein levels have been reported with worsening liver function [38] and a correlation with poor outcomes, independent of commonly used prognostic scores like Child–Pugh or MELD [39,40]. Association between poor outcomes, including delayed and/or incomplete recovery of kidney function, and C-reactive protein levels has also been reported in acute tubular necrosis [41,42].

Both cirrhosis and AKI are characterized by systemic inflammation, which adversely affect not only the liver and kidneys but also cardiac function. Since in our patients with non-HRS-AKI, both C-reactive protein at admission and history of heart failure were independently associated with incomplete recovery of renal function, it would be tempting to speculate that serum C-reactive protein could be used as a biomarker of liver–kidney–heart cross-talk in cirrhosis. However, given the retrospective and observational design of our study, as well as the small number of cases, we believe that these associations must be interpreted as prognostic rather than mechanistic. In addition, data from the literature do not consider C-reactive protein as a reliable marker of liver–kidney–heart interaction in cirrhosis due to the high variability in serum levels depending on the severity of liver disease or the occurrence of complications such as bleeding or bacterial infection [43]. In our study, we analyzed the severity of cirrhosis only at the time of admission, and complications were recorded only at the time of admission, and only if they were the cause of AKI; death and the degree of recovery of renal function could have been influenced by numerous complications during hospitalization. As a result, taking into consideration our findings and given that C-reactive protein is inexpensive, readily available, and routinely measured at hospital admission, it may be used for early risk stratification in patients with non-HRS-AKI, identifying individuals at increased risk of both in-hospital mortality and incomplete renal recovery.

### Study Limitations

This study has several limitations.

First, its retrospective single-center design and the referral pattern of our emergency institution may have introduced selection bias and may limit the generalizability of the findings. Because the referral source and mode of hospital admission were not systematically recorded, we could not quantify the proportion of transferred patients or assess whether transferred and directly admitted patients differed in mortality. Therefore, the potential contribution of referral patterns to the observed mortality cannot be excluded.

Second, the retrospective design may have resulted in missing or inaccurately documented clinical data. Detailed heart failure phenotyping, including LVEF, HFrEF/HFpEF classification, and NYHA functional class, was not systematically available and therefore could not be incorporated into the analysis. Also, several potentially relevant clinical variables, including systolic blood pressure, ascites severity, hepatic encephalopathy, active alcohol use, alcohol-associated hepatitis, and exposure to vasoconstrictors or albumin, were not systematically available for the entire cohort and therefore could not be evaluated as independent predictors. Similarly, medication exposures were not systematically captured as independent covariates, although diuretic and nephrotoxic exposure was considered when determining the presumed etiology of AKI.

Third, the relatively small sample size limited statistical power, particularly for subgroup analyses. Although the number of outcome events allowed multivariable modelling, the limited sample size, especially for the renal recovery analysis, may have increased the risk of model overfitting. Consequently, the identified predictors should be interpreted with caution and require external validation in larger prospective multicenter cohorts.

Fourth, classification of the AKI phenotype at admission may have been affected by some degree of misclassification, as urinary indices were unavailable in all patients, and, even when available, have limited diagnostic accuracy in patients with cirrhosis. In addition, HRS exclusion was based on the documented discharge diagnosis and corresponding ICD-10 coding rather than independent retrospective re-adjudication according to contemporary HRS-AKI diagnostic criteria; therefore, some degree of misclassification between HRS-AKI and non-HRS-AKI cannot be completely excluded.

Finally, renal recovery was assessed only at hospital discharge, and the timing of discharge varied among patients. Because kidney function may continue to improve after discharge, some patients classified as having incomplete recovery at discharge may have subsequently achieved further or complete renal recovery. Conversely, the absence of post-discharge follow-up prevented assessment of whether incomplete recovery persisted over time. Thirty- and ninety-day renal outcomes were not available in our retrospective dataset. Therefore, the renal recovery findings should be interpreted as predictors of kidney function status at hospital discharge rather than long-term renal recovery. In addition, renal recovery was analyzed only among patients who survived to hospital discharge. Because death precludes assessment of renal recovery and was itself associated with several measures of disease severity, this survivor-only analysis may be subject to survivorship bias. A competing-risk or longitudinal time-to-event analysis was not feasible because the retrospective dataset did not contain sufficiently granular information on the timing of renal recovery and post-discharge outcomes.

Despite these limitations, the study also has important strengths. It specifically focused on non-HRS-AKI, a clinically relevant but insufficiently investigated subgroup of cirrhosis-associated AKI, and evaluated only variables routinely available at hospital admission, making the proposed predictors readily applicable in daily clinical practice. The findings and their implications should be discussed in the broadest context possible. Future research directions may also be highlighted.

## 5. Conclusions

In this retrospective cohort of patients with cirrhosis-associated non-HRS-AKI, short-term outcomes appeared to be driven by different but complementary mechanisms. In-hospital mortality was primarily associated with the severity of liver disease and AKI, whereas incomplete renal recovery among hospital survivors was independently associated with pre-existing heart failure. Admission C-reactive protein was the only variable independently associated with both outcomes. These findings suggest that admission C-reactive protein deserves further evaluation as a potential risk stratification marker in prospective studies.

## Figures and Tables

**Table 1:** Baseline characteristics of the study population.

Parameter	Results
Gender	29 women, 110 men
Age—years	65 (36–86; IQR 14)
Comorbidities	Diabetes—42 (30.21); HT—65 (46.76); CAD—72 (51.79); heart failure—45 (32.37); pulmonary disease—16 (11.51); stroke history—16 (11.51); active neoplasia—26 (18.70)
Etiology of cirrhosis	Alcohol-associated—96 (69.06); chronic viral hepatitis—14 (10.07); NASH—9 (6.47); cardiac—14 (10.07); other or unknown—6 (4.31)
Cirrhosis Child–Pugh class	A—28 (20.14); B—46 (33.09); C—65 (46.76)
MELD-sodium score	26 (8–46; IQR 14)
AKI without pre-existing CKD	69 (49.64)
AKI superimposed on pre-existing CKD	70 (50.36)
AKI KDIGO stages	1—61 (43.88); 2—33 (23.74); 3—45 (32.37)
Etiology of AKI	Prerenal—78 (56.11); intrinsic—61 (43.88)
Length of hospital stay—days	7 (1–37; IQR 9)
Deaths	57 (41)
ICU admission	27 (19.42)
Recovery of kidney function in survivors (*n* = 82)	Complete recovery—35 (42.68)
	Incomplete recovery—47 (57.31)

Continuous variables are presented as median (IQR; range), and categorical variables as *n* (%). AKI, acute kidney injury; CAD, coronary artery disease; ICU, intensive care unit; KDIGO, Kidney Disease: Improving Global Outcomes; MELD-Na, Model for End-stage Liver Disease-Sodium; NASH, non-alcoholic steatohepatitis.

**Table 2:** Etiology of non-HRS-AKI.

AKI Type	Main Subtype	*n* (%)
Prerenal (*n* = 78)	Volume depletion	53 (64.94)
	Reduced cardiac output	6 (7.69)
	Drug-induced *	10 (12.82)
	Sepsis-associated **	9 (11.53)
Intrinsic (*n* = 61)	Ischemic ATN	19 (31.14)
	Toxic ATN	6 (9.83)
	ATIN	3 (4.91)
	Sepsis-associated ***	27 (44.26)
	Others or unknown	6 (9.83)

* Drug-induced: NSAIDs, ACE-inhibitors/ARBs, or other agents causing hemodynamically mediated reduction in glomerular filtration, without structural tubular injury (diuretics were excluded); ** mechanism was hypovolemia and/or hypotension secondary to sepsis; *** sepsis-associated ATN. AKI, acute kidney injury; ATN, acute tubular necrosis; ATIN, acute tubulointerstitial nephritis.

**Table 3:** Comparison between survivors and non-survivors.

Variable	Non-Survivors (*n* = 57)	Survivors (*n* = 82)	*p*
**Patient characteristics**			
Age—years	63 (36–82; IQR 15)	67 (37–86; IQR 15)	0.016
Gender	9 women; 48 men	20 women; 62 men	0.220
Diabetes	12 (21.05)	30 (36.58)	0.05
Hypertension	21 (36.8)	44 (53.7)	0.051
CAD	29 (50.87)	43 (52.43)	0.856
Heart failure	18 (31.57)	27 (32.92)	0.867
Advanced pulmonary disease	6 (10.52)	10 (12.19)	0.762
Stroke history	8 (14.03)	8 (9.75)	0.437
Active neoplasia	11 (19.29)	15 (18.29)	0.881
Previous CKD	18 (31.57)	52 (63.41)	<0.001
**Cirrhosis**			
MELD-sodium score	32 (15–46; IQR 10.5)	20 (8–38; IQR 13)	<0.001
Child–Pugh class	A = 5 (8.77)	A = 23 (28.04)	<0.001
	B = 9 (15.78)	B = 37 (45.12)	
	C = 43 (75.43)	C = 22 (26.82)	
Etiology	Alcohol = 40 (70.17)	Alcohol = 56 (68.29)	0.595
	Viral = 8 (14.03)	Viral = 6 (7.31)	
	NASH = 3 (5.26)	NASH = 6 (7.31)	
	Cardiac = 4 (7.01)	Cardiac = 10 (12.19)	
	Others = 2 (3.50)	Others = 4 (4.87)	
**AKI phenotype at admission**			
AKI KDIGO stages	Stg. 1 = 6 (10.52)	Stg. 1 = 55(67.07)	<0.001
	Stg. 2 = 18 (31.57)	Stg. 2 = 15(18.29)	
	Stg. 3 = 33 (57.89)	Stg. 3 = 12(14.63)	
Etiology of AKI			<0.001
Prerenal	10 (17.54)	68 (82.92)	
Intrinsic	47 (82.45)	14 (17.07)	
Sepsis etiology of AKI (both prerenal and intrinsic)	23 (40.35)	13 (15.85)	0.001
**In-hospital data**			
Length of hospital stay—days	5	8	0.109
ICU admission	26 (45.6)	1 (1.2)	<0.001
Dialysis need during hospitalization	14 (24.56)	7 (8.53)	0.009
**Median laboratory data at admission**			
Hemoglobin, g/dL	10 (3.7–16.7; IQR 3.5)	9.45 (4–18.8; IQR 3.9)	0.603
Albumin, g/dL	2.56 (0.69–3.94; IQR 0.865)	3.05 (0.55–4.72; IQR 0.96)	<0.001
Urea, mg/dL	96.10 (18.2–334.7; IQR 100)	92.30 (28–216; IQR 43.8)	0.942
CRP, mg/L	70 (2.25–237.6; IQR 72.65)	38.75 (0.61–277.4; IQR 33.41)	<0.001
Platelets, ×10^3^	132 (20–524; IQR 153)	136 (10–263; IQR 91)	0.905
Sodium, mEq/L	128.7 (114–153; IQR 9)	136 (125–143; IQR 6.6)	<0.001

Continuous variables are presented as median (IQR; range), and categorical variables as *n* (%). AKI, acute kidney injury; CAD, coronary artery disease; CKD, chronic kidney disease; CRP, C-reactive protein; ICU, intensive care unit; KDIGO, Kidney Disease: Improving Global Outcomes; MELD-Na, Model for End-stage Liver Disease-Sodium; NASH, non-alcoholic steatohepatitis.

**Table 4:** Univariable logistic regression analysis of predictors of in-hospital mortality.

Variable	OR	95% CI	*p*
Age	0.963	0.933–0.994	0.018
Hypertension	0.50	0.25–1.01	0.052
Diabetes	2.16	0.992–4.72	0.052
Previous CKD	0.266	0.130–0.545	<0.001
Child–Pugh class			
B vs. A	1.119	0.3334–3.755	0.856
C vs. A	8.991	3.0075–26.878	<0.001
MELD-sodium (per 1 point increase)	1.218	1.14–1.30	<0.001
KDIGO—AKI stage			
2 vs. 1	11.00	3.71–32.58	<0.001
3 vs. 1	25.20	8.64–73.54	<0.001
Intrinsic AKI vs. prerenal AKI	22.829	9.350–55.736	<0.001
Need for acute HD during hospitalization	3.488	1.307–9.310	0.013
Sepsis	3.590	1.622–7.947	0.002
Albumin	0.340	0.196–0.590	<0.001
CRP (per 10 mg/L increase)	1.138	1.051–1.232	0.001

AKI, acute kidney injury; CKD, chronic kidney disease; CRP, C-reactive protein; HD, hemodialysis; KDIGO, Kidney Disease: Improving Global Outcomes; MELD-Na, Model for End-stage Liver Disease-Sodium.

**Table 5:** Full multivariable logistic regression models for in-hospital mortality.

Predictor	Model A	Model B	Model C	Model D
**MELD-Na, per 1-point increase**	1.158 (1.069–1.256); *p* < 0.001	1.160 (1.071–1.257); *p* = 0.001	1.116 (1.019–1.222); *p* = 0.027	1.127 (1.027–1.236); *p* = 0.012
**KDIGO stage 2 vs. 1**	4.228 (1.149–15.563); *p* = 0.030	4.184 (1.183–14.797); *p* = 0.026	3.367 (0.808–14.021); *p* = 0.095	3.364 (0.793–14.268); *p* = 0.100
**KDIGO stage 3 vs. 1**	6.707 (1.728–26.042); *p* = 0.006	5.711 (1.510–21.604); *p* = 0.010	2.456 (0.509–11.851); *p* = 0.263	2.363 (0.480–11.626); *p* = 0.290
**Pre-existing CKD, yes vs. no**	0.233 (0.085–0.644); *p* = 0.005	0.248 (0.093–0.664); *p* = 0.005	0.133 (0.038–0.462); *p* = 0.001	0.140 (0.040–0.492); *p* = 0.002
**Admission CRP, per 10 mg/L increase**	1.118 (1.014–1.234); *p* = 0.025	—	—	1.074 (0.961–1.201); *p* = 0.212
**Sepsis, yes vs. no**	—	2.305 (0.817–6.502); *p* = 0.115	—	1.217 (0.354–4.181); *p* = 0.755
**Intrinsic AKI, yes vs. no**	—	—	15.524 (4.124–58.430); *p* < 0.001	12.446 (3.204–48.339); *p* < 0.001

Values are adjusted odds ratios (ORs), with 95% confidence intervals in parentheses. CRP, C-reactive protein; AKI, acute kidney injury; CKD, chronic kidney disease. Model A was the primary parsimonious model. Models B–D were prespecified sensitivity models examining the independent contribution of sepsis and/or intrinsic AKI. CRP was scaled per 10 mg/L increase. ORs represent adjusted odds of in-hospital mortality.

**Table 6:** Comparison between patients with complete recovery (*n* = 35 cases) and incomplete recovery (*n* = 47 cases) of renal function.

Parameter	Complete Recovery (*n* = 35)	Incomplete Recovery (*n* = 47)	*p*
**Patient characteristics**			
Age—years	64 (37–86; IQR 13)	70 (39–85; IQR 13)	0.097
Gender	9 women; 26 men	11 women; 36 men	0.810
Diabetes	13 (37.14)	17 (36.17)	0.928
Heart failure	7 (20.00)	20 (42.55)	*0.032*
Coronary artery disease	16 (45.71)	27 (57.44)	0.293
Advanced pulmonary disease	5 (14.28)	5 (10.63)	0.618
History of stroke	3 (8.57)	5 (10.63)	0.755
Hypertension	16 (45.71)	28 (59.57)	0.213
Active neoplasia	6 (17.14)	9 (19.14)	0.816
Previous CKD	20 (57.14)	32 (68.08)	0.309
Previous CKD patients (*n* = 52)			0.041
eGFR 60–45 mL/min/1.73 m^2^	12	10	
eGFR < 45 L/min/1.73 m^2^	8	22	
**Cirrhosis**			
MELD-Na score	18 (8–33; IQR 15)	20 (11–38; IQR 12)	0.179
Child–Pugh class	A = 8 (22.85)	A = 15 (31.91)	0.015
	B = 22 (62.85)	B = 15 (31.91)	
	C = 5 (14.28)	C = 17 (36.17)	
Etiology of cirrhosis	Alcohol = 26 (74.28)	Alcohol = 30 (63.82)	0.654
	Viral = 3 (8.57)	Viral = 3 (6.38)	
	NASH = 1 (2.85)	NASH = 5 (10.63)	
	Cardiac = 4 (11.42)	Cardiac = 6 (12.76)	
	Others = 1 (2.85)	Others = 3 (6.38)	
**AKI**			
AKI stages (KDIGO)	Stg. 1 = 29 (82.85)	Stg. 1 = 26 (55.31)	0.014
	Stg. 2 = 2 (5.71)	Stg. 2 = 13 (27.65)	
	Stg. 3 = 4 (11.42)	Stg. 3 = 8 (17.02)	
Etiology of AKI			<0.001
Prerenal	35 (100)	33 (70.21)	
Intrinsic	0 (0.00)	14 (29.78)	
Sepsis etiology of AKI (both prerenal and intrinsic)	4 (11.42)	9 (19.14)	0.344
In-hospital data			
Length of hospital stay—days	7 (2–24; 6)	8 (1–34; 6)	0.021
Need for HD during hospitalization	0	7 (14.89)	0.017
**Median laboratory data at admission**			
Hemoglobin, g/dL	10.3 (4–18.8; IQR 3.4)	8.9 (6.2–15; IQR 4.1)	0.103
Urea, mg/dL	92.3 (41.8–216; IQR 78.8)	92.3 (28–200; IQR 46.2)	0.722
CRP, mg/L	17.7 (0.61–117; IQR 46.11)	43.8 (10.52–277.4; IQR 30)	0.004
Albumin, g/dL	3.19 (1.06–4.72; IQR 1.14)	2.84 (0.55–4.38; IQR 0.9)	0.044
Platelet count, ×10^3^	157 (31–256; IQR 58)	111 (10–263; IQR 98)	0.072
Sodium, mEq/L	137 (125–143; IQR 11.4)	136 (125.9–143; IQR 5.6)	0.619

Continuous variables are presented as median (IQR; range), and categorical variables as *n* (%). AKI, acute kidney injury; CKD, chronic kidney disease; CRP, C-reactive protein; KDIGO, Kidney Disease: Improving Global Outcomes; MELD-Na, Model for End-stage Liver Disease-Sodium.

**Table 7:** Univariable logistic regression analysis of factors associated with incomplete renal recovery at hospital discharge.

Variable	OR	95% CI	*p*
Age	1.027	0.9848–1.07	0.214
Heart failure	2.963	1.079–8.14	0.035
Previous GFR < 45 mL/min/1.73 m^2^	3.300	1.029–10.59	0.045
Child–Pugh class			
B vs. A	0.364	0.123–1.07	0.066
C vs. A	1.813	0.487–6.76	0.375
KDIGO—AKI stage			
2 vs. 1	7.250	1.493–35.20	0.014
3 vs. 1	2.231	0.601–8.28	0.231
Platelet count	0.994	0.987–1.00	0.109
Albumin	0.498	0.257–0.965	0.039
CRP (per 10 mg/L increase)	1.172	1.000–1.344	0.046

AKI, acute kidney injury; CRP, C-reactive protein; GFR, glomerular filtration rate; HD, hemodialysis; KDIGO, Kidney Disease: Improving Global Outcomes.

**Table 8:** Multivariable logistic regression analysis of factors independently associated with of incomplete recovery of renal function at hospital discharge.

Variable	Adjusted OR (95% CI)	*p* Value
Heart failure	3.071 (1.095–8.62)	0.033
CRP at admission (per 10 mg/L increase)	1.17 (1.00–1.34)	0.047

CRP, C-reactive protein.

## Data Availability

The datasets generated and/or analyzed during the current study are available from the corresponding author on reasonable request, subject to approval by the institutional ethics committee and applicable data protection regulations.
